# One More Disguise in the Stealth Behavior of *Streptococcus pyogenes*

**DOI:** 10.1128/mBio.00661-16

**Published:** 2016-05-17

**Authors:** Vincent A. Fischetti, James B. Dale

**Affiliations:** a The Rockefeller University, Laboratory of Bacterial Pathogenesis and Immunology, New York, New York, USA; b University of Tennessee Health Science Center, Department of Medicine, Memphis, Tennessee, USA

## Abstract

The ability to hide in the animal kingdom is essential for survival; the same is true for bacteria*. Streptococcus pyogenes* is considered one of the more successful stealth bacteria in its production of a hyaluronic acid capsule that is chemically identical to the hyaluronic acid lining human joints. It has also acquired the capacity to enter eukaryotic cells to avoid the onslaught of the host’s immune defenses, as well as drugs. From this intracellular vantage point, it may remain dormant from days to weeks, only to cause disease again at a later time, perhaps causing a relapse in a drug-treated patient. We now learn that it is able to enter macrophages as well, enabling the *Streptococcus* to use this “Trojan horse” approach to be transported to distant sites in the body.

## COMMENTARY

*Streptococcus pyogenes* (group A *Streptococcus*) is a major human pathogen that causes a wide array of clinical syndromes, ranging from uncomplicated infections of the pharynx and skin to more serious infections, such as necrotizing fasciitis and toxic shock (1). *S. pyogenes* infections are, for the most part, human specific, and thus far, no reservoir besides humans has been identified. Additionally, the species comprises more than 120 different M/*emm* types that have somehow managed to be maintained in the human population. The classic view has been that acute infections eventually result in asymptomatic carriage of the organism on mucosal or skin surfaces, a process that permits survival of the species over time. Indeed, studies have shown that, at any given time, 5 to 25% of humans (depending on the time of year and the age of the person) are colonized with *S. pyogenes* (2). Exactly how the organisms maintain a presence in an otherwise hostile environment and generate new *emm* types has never been quite clear.

Now, recent studies, including the recent report in *mBio* by O’Neill et al. (3), have shown that *S. pyogenes*, which has typically been considered an extracellular pathogen, has the capacity to invade host cells and persist in the intracellular environment. *S. pyogenes* has evolved mechanisms to invade and persist in epithelial cells (4), neutrophils (5), and macrophages (6). Osterlund et al. (4, 7) originally provided clinical evidence of streptococcal tonsil cell internalization, reporting the presence of viable intra- and extracellular streptococci in excised tonsils by using electron microscopy and immunohistochemistry. Convincingly, they found intracellular *S. pyogenes* in pharyngeal epithelial cells in 13 (93%) of 14 patients with tonsillitis. Furthermore, intracellular *S. pyogenes* was found in macrophage-like cells in eight (73%) tonsils and in epithelial cells in four (36%) tonsils from 11 asymptomatic *S. pyogenes* carriers. The organism essentially commandeers the host cells as shelters from innate defenses and even antibiotics (8). The mechanisms used to thwart the microbicidal defenses of the intracellular environment of the macrophage were detailed by Wessels et al. (6), who showed that the partnership between streptolysin O (SLO) and NADase resulted in the inability of the phagolysosome to properly acidify, creating an environment that favors bacterial survival. Colonizing *S. pyogenes* has also been shown to develop mutations that affect capsule expression, resulting in increases in both adhesion and internalization into host pharyngeal cells (9). In general, such strains are avirulent or poorly virulent; however, a recently described acapsular type *emm89 S. pyogenes* strain has been shown to cause invasive disease (10). This strain exhibits increased expression of SLO and NADase, enhancing internalization and intracellular survival. The recombination events that produce this strain were found to be extensive, again emphasizing the unknown nature of the environment fostering such events.

In their recent report, O’Neill et al. (3) have confirmed the role of SLO in the process of cellular invasion and persistence and also extended the observations by showing that *S. pyogenes* can actually propagate within the cytoplasm of macrophages. Rather than rely on aggregate data to quantitate surviving *S. pyogenes*, the investigators observed single cells and enumerated individual intracytoplasmic cocci to confirm bacterial growth. A key finding was that the majority of surviving *S. pyogenes* bacteria formed long chains within the cell. Previous studies reporting “persistence” of *S. pyogenes* may have been influenced by the fact that one very long chain of streptococci or a pair of cocci will each yield 1 CFU, resulting in underestimation of the actual number of bacteria present. Long chaining of *S. pyogenes* is known to occur in response to environmental stress, such as reduced pH and nutrient depletion (11). The results reported by O’Neill et al. add to the proposition that the intracellular compartment is a place of silent sequestration for *S. pyogenes* and perhaps other, similar, pathogens.

Coincidentally, in a recent publication (12), Nelson and coworkers discovered that a phage lysin, PlyC, which is specific for *S. pyogenes* and a few other related streptococci, is able to actively penetrate eukaryotic cells inhabited by *S. pyogenes* and kill the otherwise concealed bacteria. In their studies, they tested human alveolar and primary epithelial cells and tonsil epithelial cells but not macrophages. This suggests that intracellular residency is not a fail-safe lifestyle for *S. pyogenes* and that therapies may also be manipulated to kill these intracellular bacteria.

Taken together, these studies describe the complex mechanisms involved in the host-pathogen relationship that are likely critical for the long-term survival of *S. pyogenes* in the human population. The tissue macrophage is a key component of the innate immune response to pathogenic microbes. However, it appears that virulence determinants of *S. pyogenes* have evolved to at least partially circumvent the antibacterial function of these first responders by creating a protected intracellular environment where they not only persist but actually grow. By doing so, they could survive for weeks, or even months, at the site of the original infection, evade the action of antibiotics (8), and retain the ability to be transmitted to other hosts. In some cases, this could also explain recurrent symptomatic infections with the same serotype of *S. pyogenes*, a clinical scenario that continues to perplex health care providers (4). In addition, it is interesting to speculate that *S. pyogenes* may transform the macrophage into a “Trojan horse” harboring virulent bacteria that are transported from the pharynx or tonsils to distant anatomical sites and cause deep tissue infections. This may be the mechanism behind the significant number of cases of necrotizing fasciitis that present without a focus of infection elsewhere or a potential portal of entry through the skin (13). Continued efforts to define the host-pathogen interplay may lead to novel interventions, such as phage lysins (12) or small-molecule antimicrobials that penetrate the host cell and kill stealthy streptococci, thus thwarting their highly evolved strategy.
